# Accumulation of lysosulfatide in the brain of arylsulfatase A-deficient mice

**DOI:** 10.1186/1476-511X-10-28

**Published:** 2011-02-07

**Authors:** Maria Blomqvist, Volkmar Gieselmann, Jan-Eric Månsson

**Affiliations:** 1 Department of Psychiatry and Neurochemistry, Institute of Neuroscience and Physiology, University of Gothenburg, Sweden; 2 Institute fur Physiologische Chemie, Universität Bonn, Germany

## Abstract

Lysosomal storage diseases are a group of disorders where accumulation of catabolites is manifested in the lysosomes of different cell types. In metachromatic leukodystrophy (Arylsulfatase A [EC.3.1.6.8] deficiency) storage of the glycosphingolipid sulfatide in the brain leads to demyelination, resulting in neuromotor co-ordination deficits and regression. In a mouse model for metachromatic leukodystrophy, the ASA null mutant mouse, the accumulation of sulfatide in correlation to phenotype has been thoroughly investigated. Another lipid species reported to accumulate in patients with metachromatic leukodystrophy is the sulfatide related lipid lysosulfatide. Lysosulfatide was shown to be a cytotoxic compound in cell culture experiments and thus suggested to be involved in the pathology of metachromatic leukodystrophy. In this study, we further investigated the developmental profile of lysosulfatide in the brain of ASA null mutant mice by using high performance liquid chromatography. Lysosulfatide could be detected in the brain of normal mice (ASA +/+) from 1.8 months up to 23.1 months of age. From the age of 8.8 months the lysosulfatide levels remained constant at 1 pmol/mg wet tissue. The developmental change (< 20 months) of brain lysosulfatide showed an accumulation in ASA null mutant mice at ages above one month compared to its normal counterpart (ASA +/+). Thus, the ASA null mutant mouse might be a suitable model to further investigate the role of lysosulfatide in the pathogenesis of metachromatic leukodystrophy.

## Introduction

The hypothesis of lysosphingolipids being cytotoxic catabolites in leukodystrophies was put forward in 1972 by Miyatake and Suzuki [1]. They investigated globoid cell leukodystrophy (Krabbe disease), caused by a deficiency of galactosylceramidase, and found increased concentrations of galactosylsphingosine (psychosine) in post-mortem brain tissue. The accumulation of galactosylsphingosine in the Krabbe brains was confirmed by Svennerholm et al. who also structurally characterized the isolated compound [2]. An accumulation of galactosylsphingosine was also shown in the natural mouse model for Krabbe disease, the twitcher mouse [3,4], and a close relationship between the abnormal abundance of galactosylsphingosine and demyelination was suggested. In other lysosomal storage diseases (LSDs), such as Gaucher disease, GM1 and GM2 gangliosidoses, Niemann-Pick type A and Fabry disease, abnormal accumulations of lysosphingolipids in various tissues have also been shown [5-10].

**Figure 1:**
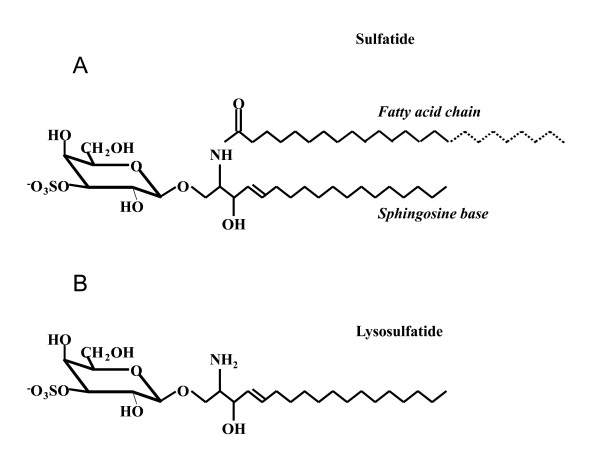
**Sulfatide (A) and lysosulfatide (B) structures**. The glycosphingolipid sulfatide (galactosylceramide-3-O-sulfate) consists of a ceramide backbone (i.e. a long-chain base and a fatty acid chain) and a sulfated galactose moiety. Lysosulfatide is the deacylated form of sulfatide (sulfogalactosylsphingosine).

Metachromatic leukodystrophy (MLD) is an autosomal recessive inherited LSD (OMIM 250100) where the enzyme arylsulfatase A (ASA, EC. 3.1.6.8) is deficient [11]. Due to this enzyme deficiency, galactosylceramide-3-O-sulfate (Sulfatide, Figure 1) accumulates within the lysosomes of various tissues. Sulfatide is a major component of the myelin sheet in the nervous system and accumulation of sulfatide in oligodendrocytes of this region leads to severe demyelination. However, sulfatide is probably not responsible for the complete demyelination process since this compound is a natural non-toxic component of brain myelin. Furthermore, the accumulation of sulfatide does not accompany the demyelination in some MLD patients [12].

Another lipid species reported to accumulate in patients with MLD is lysosulfatide (the deacylated form of sulfatide or sulfogalactosylsphingosine, Figure 1) [13,14]. Lysosulfatide is considered to be a cytotoxic substance *in vitro*, where 150 μM lysosulfatide showed a 50% inhibition of protein kinase C [15] and 50-100 μM inhibited cytochrome c oxidase activity [13]. Furthermore, lysosulfatide sulfatase is deficient in MLD fibroblasts [16]. Recent studies have also shown regulatory functions of lysosulfatide using neuronal precursor cells, where lysosulfatide inhibited cell migration via calcium-mediated process collapse [17].

The ASA null mutant mouse model created by Hess et al [18] was shown to have no ASA enzyme activity and the pattern of lipid storage resembles that of the human situation. The most severe form of MLD in humans (late infantile), which is caused by a complete lack of ASA activity, is associated with progressive demyelination, neuromotor co-ordination deficits and regression. However, the ASA null mutant mice (no ASA activity) show an attenuated phenotype compared with MLD patients with no enzyme activity. The animals display demyelination of the peripheral nervous system, astrogliosis and aberrant morphology of Bergmann glia and cerebellum [19]. Furthermore, mice become deaf and show various neuromotor abnormalities.

The aim of this study was to investigate the occurrence and developmental profile of lysosulfatide in an ASA null mutant mouse model by using high performance liquid chromatography (HPLC).

## Materials and methods

### Animals and animal tissues

Chimeric male mice obtained by injection of embryonic stem cells with targeted disruption of the ASA gene into blastocysts [18] were mated with C57BL/6J wildtype females. The offspring were analyzed by Southern blot analysis of tail DNA for the presence of the disrupted ASA allele. Mice heterozygous for the knockout allele (ASA +/-) were mated to obtain ASA-deficient (ASA -/-) and wildtype control mice (ASA +/+). Animals were bred under standard conditions and all experimental protocols were performed with due permission from the appropriate local and national authorities.

For lipid analyses, mice were anaesthetized with CO_2 _and killed by cervical dislocation. The brains were dissected out (the cerebellum and medulla oblongata were removed), immediately frozen in liquid nitrogen and stored at -80°C. ASA -/- mice were examined at 1.1, 4.2, 9.2, 15.6, 18.5 and 20.6 months of age with 2-4 animals in each group. ASA +/+ mice were examined at 1.8, 4.9, 8.8, 13.0, 16.3, 17.2 and 23.1 months of age with 1-4 animals in each group.

### Lipids

Lysosulfatide and glucosylsphingosine used as calibrators were produced and characterized at the Neurochemistry laboratory, Mölndal, Sweden [20]. The structure of synthesized lysosphingolipids was confirmed with electrospray ionization-mass spectrometry (quadrupole-time-of-flight, Micromass, Manchester, UK). Radiolabelling of lysosulfatide was performed according to the method by Schwarzmann *et al *[21].

### Lipid extraction and separation

Frozen brains were mortared under liquid nitrogen and approximately 100 mg of the homogenized brain powder was taken for lipid extraction with chloroform:methanol:water, (C/M/W 4:8:3, by vol.) [22]. From the lipid extract, an aliquot corresponding to approximately 10 mg (1/10) homogenized brain powder was evaporated and resolved in 5 mL methanol/1M KOH (9/1 by vol,). Saponification was performed at 37°C for 2 h after which 5 mL water was added.

The brain extracts were further purified on LCR-C18 columns (Varian Inc, Palo Alto, CA, USA). The LCR-C18 column was activated and washed with 10 bed volumes of methanol and C/M (1:2 by vol.) respectively, followed by 20 bed volumes of methanol and water, respectively. The sample was applied to the column and subsequently 80 bed volumes of water. Lysosulfatide was eluted with 20 bed volumes of M/W (62/38 by vol.) and an internal standard (glucosylsphingosine, 300 pmol) was then added to each sample. The sample was evaporated, resolved in 100 μL methanol and derivatized as described below.

The recovery of lysosulfatide was determined by adding ^3^H-lysosulfatide (corresponding to 830 Bq) to the lipid extract from brain tissue, as described above. The amount of ^3^H-lysosulfatide in the "lysosulfatide fraction" eluted from the LCR-C18 column was determined by scintillation counting (Perkin Elmer Tri-Carb 2800 TR, MA, US) and the recovery was 50% throughout the whole procedure (triplicates, data not shown).

### Calibrators

Calibrators for lysosulfatide quantification were prepared from stock solutions (1 nmol/50 μL methanol) and amounts of 10, 25, 50, 100, 250, 500 and 1000 pmol were used. The internal calibrator glucosylsphingosine (300 pmol) was added to each standard. The volume was corrected to 100 μL by adding methanol to each standard. Calibrators were N-derivatized with 40 μL O-phthalaldehyde-(OPA) (Pierce, Illinois, US) for 15 minutes at RT. The detection limit for lysosulfatide was approximately 2 pmol.

### Quantification of lysosulfatide

Lysosulfatide and glucosylsphingosine were N-derivatized with 40 μL OPA (Pierce) for 15 minutes at RT. Chromatography was performed by the HPLC Varian 9010 system, fitted with a Genesis C18 column (4 μm, 150 × 3 mm, Jones Chromatography, UK). The mobile phase was methanol-35 mM phosphoric acid in water (835:165 by vol.) with a flow rate of 1.0 mL min^-1^. A JASCO 821-FP spectrofluorometer with excitation at 335 nm and emission at 420 nm wavelengths was used for peak detection. Data were collected by PE NELSON chromatography software (Perkin Elmer Inc., MA, US).

### Evaluation

The amount of lysosulfatide in the samples was calculated using the respective standard curve, as described above. The quota of lysosulfatide/glucosylsphingosine (pmol) was calculated for each sample and multiplied by 2 for correction of the 50% recovery of lysosulfatide throughout the whole procedure. Values are expressed as mean ± SD.

## Results

**Figure 2:**
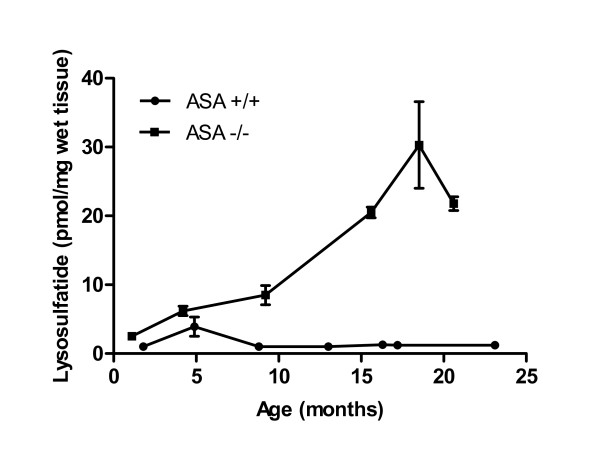
**Lysosulfatide accumulates in brain from arylsulfatase A-deficient mice**. Graphs show developmental profiles of lysosulfatide in ASA -/- mouse brains (squares) and ASA +/+ mouse brains (circles). Each age group contained 2-4 animals for ASA -/- mice and 1-4 animals for ASA +/+ mice. The mean value for each age group was calculated, as well as the corresponding SD. Error bars represents the SD of the mean.

Lysosulfatide could be detected in the brain of normal mice (ASA +/+) from the age of 1.8 months. From the age of 8.8 months, the lysosulfatide levels remained constant at 1 pmol/mg wet tissue (Figure 2). The lysosulfatide levels in the ASA -/- animals increased with age and peaked around 18.5 months of age (30 pmol/mg wet tissue) followed by a slight decline (Figure 2).

## Discussion

This investigation shows an accumulation of the lysosphingolipid lysosulfatide in the brain of Arylsulfatase A deficient mice (ASA -/-), a murine model for the human disease Metachromatic Leukodystrophy (MLD). This finding is in agreement with previous studies on post mortem brain tissues from MLD patients [13,14], where quantification of lysosulfatide used internal standards in a similar way as described above. However, one study by Rosengren et al. is not supportive [23]. The reason for this contradictory result might be the choice of method in the study by Rosengren et al., where radioimmunoaffinity TLC was used for quantification, or possibly the quality of the brain tissue.

Since the accumulation of lysosphingolipids has been observed in many of the sphingolipidoses [2,5-10], it seems reasonable that these substances might play a role in the pathogenesis of LSDs. Several studies also support this role, mainly as cytotoxic compounds. The most thoroughly investigated lyso-compound is the lysosphingolipid of galactosylceramide, psychosine, which accumulates in Krabbe disease [1,2,24]. The accumulation is closely related to the demyelination process and it is suggested to involve several mechanisms. These include regulating protein kinase C mediated growth factor, interfering with IGF-1 signalling, activating phospholipiase A2 and thus producing lysophosphatidylcholine-induced apoptosis and activating TDAG8 receptors resulting in the production of multinuclear globoid cells, reviewed by [25]. One might assume similar mechanisms being involved in the pathogenesis of MLD but this remains to be investigated. Thus, the observation of accumulated lysosulfatide in the ASA -/- mouse suggests this model to be a suitable tool for functional studies of lysosulfatide in the pathogenesis of MLD.

It must be mentioned that the ASA deficient mouse does not demyelinate in the central nervous system [18]. In this respect it is interesting to compare the lysosulfatide levels found in patients who develop demyelination. In the white matter of MLD patients, levels ranging from 50-200 pmol/mg wet weight of lysosulfatide have been found [13,14]. These levels are higher than those found in the non demyelinating mice. Furthermore, the concentrations of lysosulfatide were found to be similar when comparing human control cerebral white matter with the brain of ASA +/+ mice. Thus, if lysosulfatide is indeed a major cause of demyelination in MLD the comparatively low levels found in ASA -/- mice would explain the lack of demyelination in this animal model.

Lipid rafts, enriched in cholesterol and sphingolipids, act as scaffolds on which different signalling molecule assemble [26]. These domains play a central role in many cellular processes including membrane sorting and trafficking, signal transduction, cell growth and survival. The list of various diseases where raft formation plays an important role in disease progression continues to grow [27] and includes the possibility of these domains being involved at a cellular level in the pathogenesis of LSDs. White et al. recently showed an accumulation of psychosine in rafts isolated from the post mortem brain of human Krabbe patients and from brain and sciatic nerve of the twitcher mouse [28]. Psychosine accumulation was accompanied by increased levels of cholesterol in these domains and changes in the distribution of the raft markers flotilin-1 and caveolin-1. Furthermore, these molecular changes were associated with the inhibitory effect of psychosine on protein kinase C. The authors propose a model of psychosine involvement in raft-modulated cell functions by disrupting the raft architecture and deregulating signal activity (disorganisation of myelin components, inflammation, synaptic dysfunction, axonal defects). Since the ethiology of MLD is somewhat similar to Krabbe disease, it is reasonable to assume that raft disruption involving lysosulfatide accumulation might play a role in disease progression. However, the accumulation of lysosulfatide in rafts of MLD patients and ASA mice remains to be investigated.

## Conclusion

In agreement with the accumulation of lysosulfatide in the brain of patients with metachromatic leukodystrophy, ASA null mutant mice also showed increased amounts of this lysosphingolipid in the brain. Furthermore, a developmental increase of lysosulfatide was observed. Lysosulfatide is considered to be a possible pathogenic agent in MLD contributing to the severe demyelination occurring in these patients. However, the mechanism of involvement is not known. The ASA null mutant mice might be a suitable model to investigate the role of lysosulfatide in the pathogenesis of MLD, including the peripheral demyelination of these animals.

## Abbreviations

ASA: arylsulfatase A; HPLC: high performance liquid chromatography; LSD: lysosomal storage disease; MLD: metachromatic leukodystrophy; OPA: *O*-phthalaldehyde; Sulfatide: galactosylceramide-3-O-sulfate; Lysosulfatide: sulfogalactosylsphingosine

## Competing interests

The authors declare that they have no competing interests.

## Authors' contributions

JEM designed the study, MB analysed the data and drafted the paper and VG provided the tissue material. All the authors contributed to the interpretation and discussion of the results related to their part of the work, critically revised the paper and read and approved the final manuscript.
